# Experiences of International and British Medical Graduates in UK Core Psychiatry Training in the Northwest Deanery of England: ACross-Sectional Mixed-Methods Study

**DOI:** 10.1192/bjo.2026.11319

**Published:** 2026-06-30

**Authors:** Sampada Gadde, Talha Amanullah

**Affiliations:** 1 Mersey care NHS Trust, Liverpool, United Kingdom; 2 Merseycare NHS Trust, Liverpool, United Kingdom

## Abstract

**Aims:**

To compare Core Psychiatry training experiences in the UK of British and International Medical Graduates in the Northwest Deanery of England and identify challenges faced by each group across multiple domains.

**Methods:**

A cross-sectional mixed method survey was conducted (Electronic and Paper) amongst core psychiatry trainees in the Northwest Deanery of England. The survey consisted of multiple-choice questions and free text box. Quantitative data was analysed using descriptive statistics (Chi square, Fisher’s exact andoddsratio) to compare BMG and IMG responses while the qualitative responses were analysed thematically using the Braun and Clarke the maticanalysis.

**Results:**

48 trainees responded (IMG:28, BMG:20). Burnout was reported more frequently by IMGs than BMGs. IMGs were significantly more likely to report cultural differences, perceived preparedness to enter training. Thematic analysis identified concerns around day-to-day training, prioritising clinical workload over learning, frequent changes in placements, social isolation and disruption in community building. Moreover, concerns around inequity in pay and inconsistent on call demands across trusts were identified.

**Conclusion:**

IMGs experience a higher degree of burnout compared with their BMG counterparts, and more likely to report challenges like cultural differences, perceived preparedness to enter training. Challenges like increased workload, navigating the NHS systems, rota pressures and prioritising service provision over learning were commonly reported by both the groups.

